# Deep Learning Study of Alkaptonuria Spinal Disease Assesses Global and Regional Severity and Detects Occult Treatment Status

**DOI:** 10.1002/jimd.70042

**Published:** 2025-05-15

**Authors:** Kendall A. Flaharty, Vibha Chandrasekar, Irene J. Castillo, Dat Duong, Carlos R. Ferreira, Suzanna Ledgister Hanchard, Ping Hu, Rebekah L. Waikel, Francis Rossignol, Wendy J. Introne, Benjamin D. Solomon

**Affiliations:** ^1^ Medical Genomics Unit, Medical Genetics Branch National Human Genome Research Institute, National Institutes of Health Bethesda Maryland USA; ^2^ Human Biochemical Genetics Section, Medical Genetics Branch National Human Genome Research Institute, National Institutes of Health Bethesda Maryland USA; ^3^ Unit on Skeletal Genomics, Eunice Kennedy Shriver National Institute of Child Health and Human Development National Institutes of Health Bethesda Maryland USA

**Keywords:** Alkaptonuria, artificial intelligence, clinical genetics, deep learning, medical genomics, nitisinone, rare disease

## Abstract

Deep learning (DL) is increasingly used to analyze medical imaging, but is less refined for rare conditions, which require novel pre‐processing and analytical approaches. To assess DL in the context of rare diseases, this study focused on alkaptonuria (AKU), a rare disorder that affects the spine and involves other sequelae; treatments include the medication nitisinone. Since assessing x‐rays to determine disease severity can be a slow, manual process requiring considerable expertise, this study aimed to determine whether these DL methods could accurately identify overall spine severity at specific regions of the spine and whether patients were receiving nitisinone. DL performance was evaluated versus clinical experts using cervical and lumbar spine radiographs. DL models predicted global severity scores (30‐point scale) within 1.72 ± 1.96 points of expert clinician scores for cervical and 2.51 ± 1.96 points for lumbar radiographs. For region‐specific metrics, the degrees of narrowing, calcium, and vacuum disc phenomena at each intervertebral space (IVS) were assessed. The model's narrowing scores were within 0.191–0.557 points from clinician scores (6‐point scale), calcium was predicted with 78%–90% accuracy (present, absent, or disc fusion), and vacuum disc phenomenon predictions were less consistent (41%–90%). Intriguingly, DL models predicted nitisinone treatment status with 68%–77% accuracy, while expert clinicians appeared unable to discern nitisinone status (51% accuracy) (*p* = 2.0 × 10^−9^). This highlights the potential for DL to augment certain types of clinical assessments in rare disease, as well as identifying occult features like treatment status.

## 1 Introduction

Deep learning (DL) is increasingly employed in medical practice and research. While DL is a powerful tool, many questions remain about applications to smaller data sets, which are often the norm in rare disease research. For example, DL usually requires large amounts of training data, and for smaller data sets such as the ones available in rare genetic conditions, it is important to understand how well DL models can be finetuned and how publicly available data from more common diseases can be leveraged [1, 2, 3, 4].

Other work has explored issues related to these questions involving DL; for example, in radiology, there has been considerable effort in analyzing DL performance and providing large data sets that can be applied to other analyses [5]. While chest x‐ray image data sets are readily available, other types of radiographs remain less explored. Publicly available and annotated cervical and lumbar spine x‐ray datasets are relatively scarce, and only recently the CSXA and BUU‐LSPINE data sets released 4963 cervical and 3600 spine lumbar radiographs with annotations for each vertebra, respectively [6, 7]. These data sets focused on conditions involving lordosis or spondylolisthesis, which are more common than most genetic conditions affecting the spine.

In this study, in order to investigate the issues described above to a data set involving a rare genetic condition, DL analyses of cervical and lumbar spine radiographs of individuals with alkaptonuria (AKU) were conducted. AKU, the first human disorder described with an autosomal recessive inheritance, is a rare inborn error of metabolism, with a prevalence of ~1 in 250 000 births [8]. AKU occurs due to biallelic pathogenic variants in the *HGD* gene, resulting in deficient homogentisate 1,2‐dioxygenase activity. This enzyme is involved in the degradation of homogentisic acid (HGA); decreased enzymatic activity results in increased HGA. Sequelae include dark urine, ochronosis, cardiovascular disease (including aortic sclerosis and aortic valve stenosis), nephrolithiasis, prostate lithiasis in men, hypothyroidism, and most prominently arthritis affecting the spine and large joints (shoulders, knees, hips) [9, 10, 11]. Cervical and lumbar spine radiographs are often used as initial assessments of disease severity, but other imaging modalities, such as magnetic resonance imaging (MRI) and bone scintigraphy may also be used [12, 13]. Management is multi‐faceted; nitisinone, which inhibits HGA production, is approved for AKU treatment in Europe and is under investigation in the United States [13, 14, 15].

Motivations for the described DL applications in AKU are as follows. First, expert clinicians and clinical researchers need to manually and extensively annotate the sequelae of the spine to assess disease severity. This annotation requires expertise and is time‐consuming. Second, when assessing a specific area like a single intervertebral space (IVS), clinicians may benefit from being able to holistically consider the overall appearance of the spine and other landmarks and features visible on a radiograph. By training on entire images, DL models may similarly incorporate this type of context in their analyses. Finally, although AKU progression is continuous, manual scoring requires human annotators to label AKU severity on a discretized scale. A DL model can be trained to produce continuous values, which may better align with disease progression.

In the context of these motivations, the classifiers described in this study estimate AKU spine disease in two different ways: global severity for the cervical or lumbar spine and region‐specific severity of each IVS. As AKU progresses, the intervertebral spaces narrow, often leading to calcification and vacuum disc phenomenon [16]. Thus, a straightforward disease assessment is to predict a single global severity score for an entire radiograph. However, since each IVS contributes to the global severity (and as it may be clinically important to consider specific parts of the spine in addition to the overall severity), a multi‐label classifier was also trained to estimate the region‐specific severity at each IVS based on an entire radiograph.

While the study primarily focuses on severity, it was found that DL may be trained to detect whether a person is being treated with nitisinone. This aligns with previous publications in which DL models can detect findings occult to human experts [17, 18].

## 2 Methods

**FIGURE 1:**
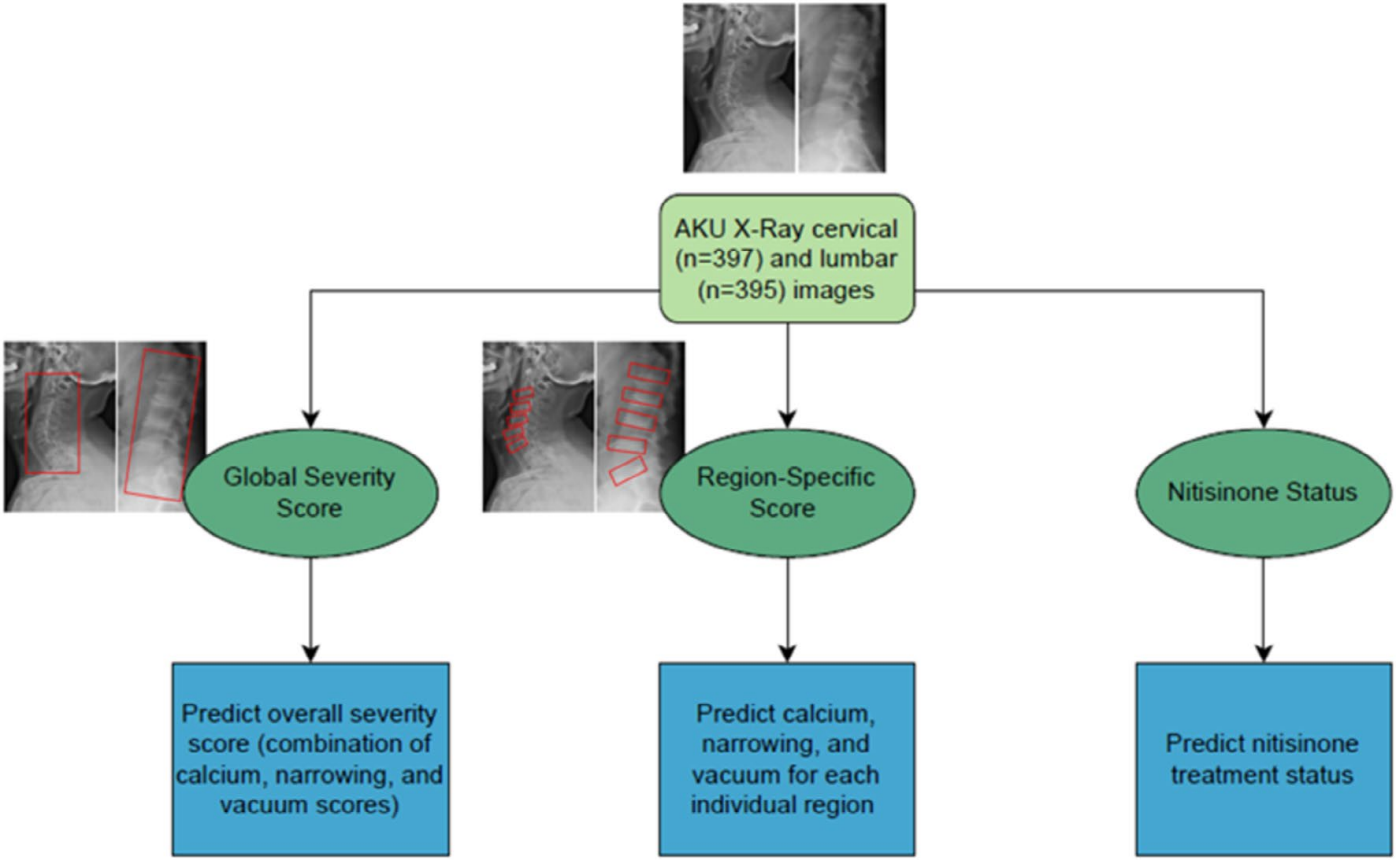
Overview of EfficientNet models trained on AKU cervical and lumbar radiographs for predicting global severity scores, region‐specific scores, and nitisinone treatment status. The red boxes in the set of radiograph images indicate the specific regions of interest analyzed to attribute scores for global severity and region‐specific severity, with these annotated areas serving as the input data used to train the model. One set of AKU images includes one lateral cervical and one lateral lumbar x‐ray taken of a patient at the same timepoint.

AKU radiographs were obtained via IRB‐approved studies at the NIH Clinical Center from January 2003 to May 2023. Demographic data of the cohort, including age, sex, nitisinone status, and number of images collected, are reported in Table 1. Of the 409 sets of lateral radiographs, 397 cervical and 395 lumbar images were analyzed in total (Figure 1, Table 1, Table S1). Neither thoracic nor anteroposterior images were analyzed due to incomplete data sets as well as advice from clinical experts regarding their interpretability (see Supporting Information). Using EfficientNet as the base neural network architecture, independent models were trained to evaluate the following: global severity, region‐specific severity, and nitisinone treatment (Figure 1) [19]. The Occlusion method was used to generate saliency maps [20]. See Supporting Information for more details about data set collection and preparation, DL model selection and implementation (i.e., optimizer, learning rate, batch size), and expert radiographic scoring criteria. The trained model weights and code are available at: https://github.com/flahartyka/AKU‐progression‐efficientnet.

**TABLE 1:** Alkaptonuria dataset demographic information and statistics.

Demographic variable	Statistic
Age range (years)	14.5–80
Mean age (years)	52.3

	Ratio	Number of images
Male:female	0.60:0.40	244:165
On:off nitisinone	0.20:0.80	80:329
Cervical mild:moderate:severe	0.63:0.24:0.13	250:95:52
Lumbar mild:moderate:severe	0.13:0.06:0.81	51:25:319
Training:testing	0.83:0.17	338:71

*Note:* Numbers of images in the dataset associated with age, sex, and nitisinone status. The grading scheme of each radiograph is the same regardless of age, sex, or nitisinone status, and demographic information is not provided to the DL model.

### 2.1 Global Severity Score

The global severity scores of an entire radiograph were calculated by summing calcium (0, 1), narrowing (0, 1, 2, 3, complete disc fusion), and vacuum disc (0, 1) scores across all IVSs, with a maximum score of 6 per IVS (maximum 30 points per image). This summation of individual IVS scores into a global severity score for each image is meant to represent a direct radiological assessment of the patient's clinical status, which can be used to estimate whether a patient has mild, moderate, or severe AKU outcomes. Higher scores indicate a greater degree of disease severity and clinical outcomes for a particular patient. See Supporting Information for additional details about this scoring system, which was based on previous studies and per ongoing current research into spinal sequelae of AKU [21]. A score of 6 was assigned for complete disc fusion at a given IVS; this is considered the maximum severity score for an IVS (i.e., calcium and vacuum disc were not graded for IVS with complete fusion).

Two classifiers were trained: one for cervical and one for lumbar images. To mimic the continuous spectrum of AKU severity, soft‐labels were used as target outputs. For each image, the ground‐truth global severity score was divided by the maximum score; thus, each image has a score ranging from 0 to 1 instead of 0 to 30. For example, an image with a total score of 20 received a 20/30 soft‐label target. The model was trained with Cross Entropy loss.

The models were evaluated on a separate test set to predict the global severity score for each radiograph, and occlusion maps were generated. For human interpretation, predicted scores were rescaled from 0 to 1 back to the 0 to 30 range by multiplying by 30, the maximum score. The average point differential from the ground truth score is reported for the entire test set, as well as for the lower 25% quartile, 25%–75% interquartile range, and upper 75% quartile of ground truth scores. Linear regression *R* ^2^ values were computed to gauge how score predictions align with ground truth labels.

### 2.2 Region‐Specific Score

Next, six classifiers were trained to estimate the IVS‐specific narrowing, calcification, and vacuum disc metrics for cervical and lumbar radiographs. In each classifier, every IVS was given its own Cross Entropy loss function.

Narrowing level (although continuous) was manually discretely labeled as 0, 1, 2, 3, or complete disc fusion (in which case, fusion was converted into a numerical score of 6, the maximum IVS‐specific score). Thus, for prediction, a weighted average was calculated to estimate the final narrowing severity level in the range of 0–6.

Calcium and vacuum disc ground‐truth labels were assigned by experts with discretized labeling: present, absent, or complete disc fusion (in which case, calcium and vacuum disc were not graded). Hence, the ground‐truth labels do not exactly reflect the continuous nature of calcium and vacuum progression. However, due to the subjectiveness during manual annotation, which is explained in the Discussion, discretized scores are used. For example, both partial and total calcification would be classified as “present”, making model categorization potentially challenging.

### 2.3 Nitisinone Status

There are no obvious indicators of nitisinone treatment identifiable by human experts in radiographs; thus, as an interesting experiment, the study aimed to determine if the model could differentiate treatment status.

The “on treatment” cohort included individuals who had been on nitisinone for at least 3 months; post‐treatment individuals (i.e., individuals who had previously been treated, but who were no longer receiving treatment) were excluded. Two EfficientNet classifiers (cervical and lumbar) were trained to classify nitisinone treatment as “on” or “off”. Each model was evaluated on the same test set described in the previous sections, and occlusion maps were generated [20].

To check whether experts could identify nitisinone status, surveys were designed using the nitisinone test images. Participants were asked whether the individual in each radiograph was receiving nitisinone (see Supporting Information for details about the survey design). Responses were compared to model predictions on the same test set.

Potential confounders such as age, time on treatment, and severity score were evaluated by comparing (via *t*‐test) between the “on” and “off” nitisinone groups.

## 3 Results

### 3.1 Global Severity Score

**FIGURE 2:**
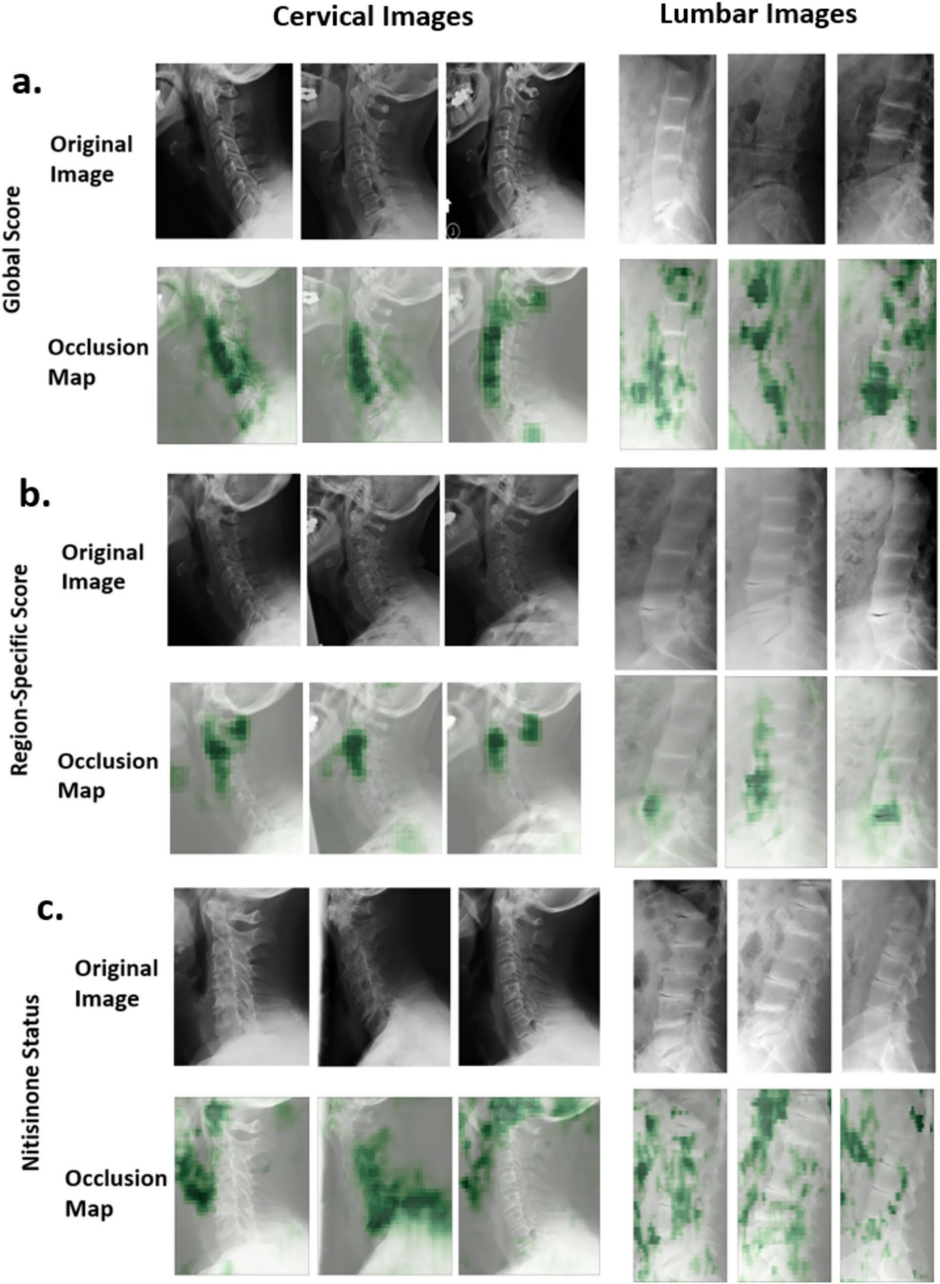
Examples of saliency maps for model prediction of (a) global scores, (b) region‐specific scores, and (c) nitisinone status on cervical and lumbar AKU radiographs. Green, shaded regions indicate regions of model attention when predicting each metric for the entire cervical or lumbar image.

An initial area of inquiry involved predicting the global severity score for cervical and lumbar radiographs (Table 2a). For all cervical images, the model obtained an average score of 1.72 ± 1.96 points from the expert scores. For lumbar images, the range was slightly wider, with an average of 2.51 ± 1.96 points from the expert scores. Figure 2a shows a few occlusion maps, along with the original images. The cervical occlusion maps tend to focus on the central neck and spine, while lumbar images have more variable foci.

**TABLE 2:** DL model predictions of (a) global (total) severity scores for the full cervical and lumbar spines, (b) narrowing, calcium, and vacuum disc status for all intervertebral spaces in the cervical and lumbar spine, and (c) nitisinone status for the full cervical and lumbar spines.

a. Global scores				
Region	Q1 (0%–25%)	Q2‐Q3 (25%–75%)	Q4 (75%–100%)	All Images
Cervical Spine	0.72 ± 0.78	1.64 ± 1.44	3.25 ± 2.86	**1.72 ± 1.96**
Lumbar Spine	3.60 ± 2.37	2.18 ± 1.64	2.04 ± 1.56	**2.51 ± 1.96**

b. Region‐specific scores			
Metric	Region	Intervertebral space	Test accuracy
Narrowing	Cervical spine	C2‐C3	0.330 ± 0.424
		C3‐C4	0.191 ± 0.292
		C4‐C5	0.270 ± 0.337
		C5‐C6	0.346 ± 0.367
		C6‐C7	0.557 ± 0.619
	Lumbar spine	L1‐L2	0.432 ± 0.369
		L2‐L3	0.394 ± 0.403
		L3‐L4	0.423 ± 0.390
		L4‐L5	0.410 ± 0.372
		L5‐S1	0.352 ± 0.376
Calcium	Cervical spine	C2‐C3	90%
		C3‐C4	85%
		C4‐C5	85%
		C5‐C6	85%
		C6‐C7	86%
	Lumbar spine	L1‐L2	84%
		L2‐L3	92%
		L3‐L4	88%
		L4‐L5	78%
		L5‐S1	82%
Vacuum disc	Cervical spine	C2‐C3	86%
		C3‐C4	90%
		C4‐C5	85%
		C5‐C6	78%
		C6‐C7	66%
	Lumbar spine	L1‐L2	55%
		L2‐L3	65%
		L3‐L4	69%
		L4‐L5	63%
		L5‐S1	41%

c. Nitisinone prediction		
Region	Intervertebral space	Test accuracy
Cervical spine	C2‐C7	83% (total) 68% (“on treatment” accuracy)
Lumbar spine	L1‐S1	87% (total) 73% (“on treatment” accuracy)

*Note:* Global scores are reported as the average point differential with the standard deviation between the DL prediction and clinical expert annotations. Narrowing status is graded using a soft label approach, assigning levels (0, 1, 2, 3, or disc fusion, which is assigned a score of 6). Narrowing scores are reported as the average point differential with the standard deviation between the DL prediction and clinical expert annotations. Calcium and vacuum disc statuses are categorized as present, absent, or fused, and reported as the accuracy of the model in making the correct prediction. Nitisinone status is categorized as “on treatment” or “off treatment”. Individuals on the drug for less than 3 months, as well as any post‐treatment images, were excluded from the cohort. Bold indicates summary values.

### 3.2 Region‐Specific Score

A second area of inquiry involved predictions of narrowing, calcium, and vacuum disc estimates for individual cervical and lumbar IVS (Table 2b). For narrowing in the cervical IVS, the model obtained a range of 0.191–0.557 points from the expert scores. Predictions for the C6–C7 IVS showed the largest score differential from expert scores. In the lumbar spine, the model achieved a consistent range of scores across all IVS (0.352–0.432 points from the expert scores).

For calcium, cervical IVS predictions obtained high accuracy (85%–90%), whereas lumbar IVS exhibited wider variability (78%–92%). For vacuum disc, accuracy ranged from 66% to 90% in cervical IVS and was less consistent (41%–69%) in lumbar IVS. The C6‐C7 and L5‐S1 IVS exhibited the lowest accuracy. Occlusion maps in Figure 2b indicate that the model tends to focus on a single IVS, rather than other unrelated areas (such as “R” or “L” metal markers).

### 3.3 Nitisinone Status

A third area of inquiry involved the prediction of nitisinone status based on cervical and lumbar images. Table 2c summarizes nitisinone status predictions in 39 and 22 images without and with treatment, respectively. For the cervical spine, the model achieved a total accuracy of 83%, with 68% accuracy for “on treatment” predictions. In the lumbar spine, performance was slightly higher, with a total accuracy of 87%, and 73% accuracy for “on treatment” predictions.

Figure 2c presents select occlusion maps and original images for nitisinone status predictions. As discussed later, these seem to focus on areas that may logically be affected by disease and treatment, including regions that involve ligaments, cartilage, and other connective tissue. Regarding potential confounders, there were no statistically significant differences across nitisinone status groups for age, sex, severity score, or time on treatment (Figure S3, Table S5).

**FIGURE 3:**
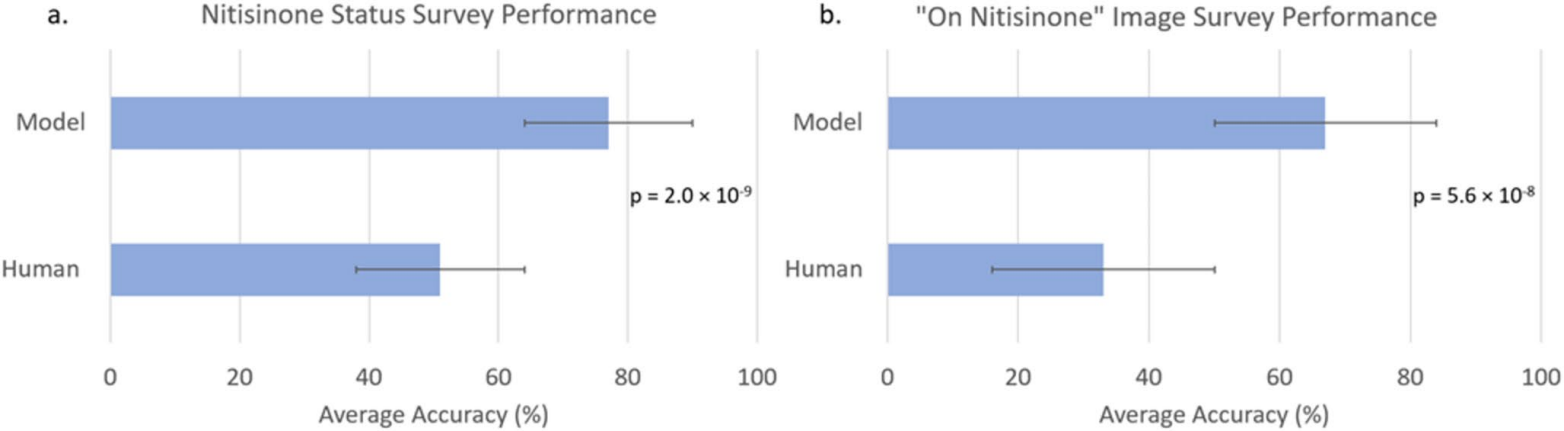
Comparison of nitisinone status detection performance between a DL model and human expert (geneticists and radiologists) on cervical and lumbar spine x‐ray images for (a) all test images (Human = 51%, Model = 77%, *n* = 61 images) and (b) “on nitisinone” only images (Human = 33%, Model = 67%, *n* = 22). Average accuracy is shown for both the model and humans, with error bars representing standard deviation. The model outperforms human expert participants, achieving significantly higher accuracy in both the entire test set and the “on nitisinone” subset (*p* = 2.0 × 10^−9^, *p* = 5.6 × 10^−8^).

The model (77% accuracy) outperformed human participants (51% accuracy) (*p* = 2.0 × 10^−9^) on a full test set of 61 images (Figure 3). When considering only the “on nitisinone” images in the surveys, the model (67%) outperformed human participants (33%) (*p* = 5.6 × 10^−8^).

## 4 Discussion

In this study, DL classifiers were trained to estimate severity scores of individuals with AKU based on cervical and lumbar radiographs. Several points are worth emphasizing.

First, we obtained varying results based on different methods, and some of our initial explorations yielded less accurate predictions. For example, as described in the Supporting Information, YOLO and SAM methods were applied to segment each IVS, and then EfficientNet was trained on each segmented IVS [1, 22]. This yielded less accurate results than training the model using entire radiographs. This may be due to high correlations among IVS within a radiograph (Tables S2 and S3). This is logical since AKU will typically affect multiple IVS [9]. Thus, even when assessing a single IVS, other IVS may provide useful information. Furthermore, other (non‐IVS) anatomical areas may correlate with spinal disease. Therefore, while segmentation methods were explored, the main analyses ultimately focused on investigating DL models with respect to the full radiographs.

When considering the full radiograph, the model's ability to predict global severity was assessed first, as this provides key information related to disease status [9]. For example, in current, ongoing clinical studies of AKU, experts can reliably correlate the radiographic scores with clinical assessments for disease progression, such as pain levels, lumbar flexibility, or other physical functioning tests, establishing these scores as valuable clinical markers of the disease progression. The DL performance metrics (based on average deviation from the ground truth) for the global severity scores are comparable to the minimal detectable change with 95% confidence interval (MDC_95_) value calculated by experts during manual annotation: 1.40 points for cervical scores and 1.61 points for lumbar scores, indicating that the models align closely with manual expert annotation. There are also strong correlations between the ground truth global severity and the predicted global severity scores (*R* ^2^ = 0.9441 for cervical and *R* ^2^ = 0.8675 for lumbar, Table S4). Given the slow‐progressing nature of AKU, the model reliably estimates overall severity within 1–2 years of disease progression (1.2–1.3 points = 1 year, per preliminary data from studies on this same data set).

The region‐specific models, while less useful in clinical practice, may align with expert clinical intuition by providing a more granular estimation of AKU‐related spine involvement. For example, expert clinicians look for a combination of different features (calcium, narrowing, and vacuum disc) in each IVS when assessing the clinical manifestations of AKU. Based on this practice, this study involved training models to do the same (Table 2a,b). The models predicted narrowing with the highest accuracy. Narrowing is evaluated on a gradient scale and can be easily observed as the physical distance between vertebrae; thus, the expert clinicians could be specific about the narrowing level, providing more training information to the model. Narrowing performs with slightly higher accuracy for the cervical than the lumbar spine. Expert clinicians suggest this may be because lumbar spine IVS are wider than cervical IVS, making relative narrowing difficult to grade in the lumbar region. Additionally, clinicians indicate that normal spines are easier to grade, and as lumbar spines are more affected, this may impact some of the results (Figure S3).

Calcium was the next best predicted metric and performs slightly better in the cervical spine. As with narrowing, this may correspond to the more severe lumbar findings. Compared to the cervical spine, the lumbar regions are more obscured by other anatomical structures, and the quality (e.g., contrast and clarity) was more disparate. Given the small data set, it is thus expected that the cervical images perform slightly better due to their uniformity.

Finally, the vacuum disc underperforms and follows the trend of higher accuracy in the cervical versus lumbar region. The vacuum disc was more subjective for expert clinicians to grade, particularly in the lumbar spine. Clinicians described needing to adjust the contrast on radiographs to assess the vacuum disc. Severe narrowing affects both vacuum disc and calcium visibility, leading to more non‐uniform appearances for both metrics in the lumbar spine, making it difficult to build a training data set.

Related to the grading subjectiveness, the regions near the edges of the images (C6‐C7, L5‐S1) usually performed with lower accuracy. The outer areas tended to be less uniform and more obscured, often affected by overlapping anatomical structures (Figure S4). Therefore, clinicians were often unable to decisively grade these regions; images without scores were removed, reducing the sample size (Table S1). Furthermore, spine angulation can vary widely depending on a person's posture, which can affect IVS appearance. In such cases, it can be hard to classify the images using small sample sizes.

While the global severity score may be a more clinically useful metric of disease progression in AKU, the region‐specific analyses provide more context to help understand global scores. Together, these results suggest novel ways that global and regional models may provide a more complete picture of AKU severity. Additionally, the relative importance of global versus regional severity may differ in other disease processes, such as conditions that manifest more focally; thus, it may be important for DL models to be able to consider both approaches [23].

The final analysis explored the model's ability to identify nitisinone status. This was particularly intriguing and suggests novel model capabilities because clinical experts have not identified radiographic signs that could reliably indicate this. Similar to findings in other studies—such as DL models predicting sex from retinal images—the results suggest that models may be able to detect patterns beyond human perception [17].

A fascinating aspect of the nitisinone analysis involved the use of occlusion maps, as mentioned above. In cervical images, the model often highlighted the laryngeal region, ligaments, cartilage, and other connective tissue (Figure 2). In lumbar images, the focus was less consistent but included areas such as the aortic region, ligaments, and cartilage. These findings are potentially consistent with nitisinone affecting HGA deposition in collagen‐rich regions, which could change the calcification of tissues in a subtle way detected by the model (but not humans). Although the saliency maps highlight regions such as the larynx, aortic region, and other connective tissues, none of the survey participants wrote comments about noticing these regions (see Supporting Information) [24].

Finally, it is important to note that this study has limitations, including the small data set. Additionally, the radiographs were collected over a 20‐year period, during which variability in imaging techniques and equipment could yield inconsistencies. For example, lumbar images varied in terms of features like contrast, clarity, and resolution. This could pose challenges for the model in recognizing subtle features. Other limiting factors included the use of discretized scores by experts who manually graded the radiographs, as disease progression is continuous; the inherent subjectivity of human assessments suggests the benefit of standardized grading protocols or finer grading scales. This study also analyzed plain x‐rays; however, other imaging techniques, such as MRI and bone scintigraphy, are also effective and reliable at evaluating AKU severity. Future work would focus on assessing machine‐learning approaches applicable to these modalities. Additional limitations are discussed in the Supporting Information.

Despite these limitations, the study highlights the promise of DL in identifying clinically relevant patterns in rare diseases and small data sets, as well as identifying features that may not otherwise be recognizable by the human eye.

## Author Contributions

K.A.F., V.C., D.D., R.L.W., P.H., S.L.H., and B.D.S. designed the experiments. I.J.C., F.R., and W.J.I. collected the data. K.A.F., V.C., and D.D. conducted the experiments. K.A.F., V.C., D.D., P.H., C.R.F., R.L.W., and S.L.H. conducted analyses of the data. K.A.F., D.D., P.H., S.L.H., R.L.W., C.R.F., F.R., W.J.I., and B.D.S. wrote the paper. K.A.F., I.J.C., D.D., C.R.F., P.H., S.L.H., R.L.W., F.R., W.J.I., and B.D.S. critically revised and approved the final paper.

## Ethics Statement

Applicable portions of the study have been approved by the following NIH IRB#: 00HG0141, 05HG0076, 000547, and 002349, and appropriate consent was obtained from all research participants in accordance with the approved protocols. These NIH IRB protocols correspond to the original 20‐year clinical trial of individuals with AKU (00HG0141), the clinical trial of individuals on nitisinone treatment (05HG0076), the analyses done in this study including using deep learning to analyze these images (000547), and the survey exemption protocol to send out surveys of these images to clinical geneticists and radiologists (002349). This article does not contain any studies with animal subjects performed by any of the authors.

## Consent

All procedures followed were in accordance with the ethical standards of the responsible committee on human experimentation (institutional and national) and with the Helsinki Declaration of 1975, as revised in 2000 (5). Informed consent was obtained from all patients for being included in the study. Patient consent was obtained with the approved protocols NIH IRB#: 00HG0141, 05HG0076. The NIH IRB provided a waiver of consent for protocols NIH IRB# 000547 and 002349.

## Conflicts of Interest

Benjamin D. Solomon is the Co‐Editor‐in‐Chief of the American Journal of Medical Genetics and receives textbook royalties from Wiley. All other authors declare no conflicts of interest.

## Supporting information


**Data S1.**jimd70042‐sup‐0001‐Supplementary_Material.

## Data Availability

Data and code availability are described in the manuscript and can be found at: https://github.com/flahartyka/AKU‐progression‐efficientnet. The original data set of AKU radiographs is not released for patient privacy reasons and related to IRB requirements. If readers are interested in accessing the AKU data set, they can contact the investigators of the clinical trial (00HG0141/NCT00005909), some of whom are co‐authors on this study.
